# Home phototherapy for neonatal hyperbilirubinemia: current practices and attitudes

**DOI:** 10.1038/s41390-024-03754-8

**Published:** 2024-12-13

**Authors:** Maryse C. Cnossen, Jessie Spaan, Maria S. Fleischmann, Berthe A. M. van der Geest, Erwin Ista, Christian V. Hulzebos, Jasper V. Been, Hanneke W. Harmsen van der Vliet – Torij

**Affiliations:** 1 Research Center Innovations in Care, Rotterdam University of Applied Sciences, Rotterdam, The Netherlands; 2 Division of Neonatology, Department of Neonatal and Pediatric Intensive Care, Erasmus MC Sophia Children’s Hospital, University Medical Center Rotterdam, Rotterdam, The Netherlands; 3 Division of Pediatric Intensive Care, Department of Neonatal and Pediatric Intensive Care, Erasmus MC Sophia Children’s Hospital, University Medical Center Rotterdam, Rotterdam, The Netherlands; 4 Division of Nursing Science, Department of Internal Medicine, Erasmus MC, University Medical Center Rotterdam, Rotterdam, The Netherlands; 5 Division of Neonatology, Department of Pediatrics, Beatrix Children’s Hospital, University Medical Center Groningen, Groningen, The Netherlands; 6 Department of Obstetrics and Gynecology, Erasmus MC Sophia Children’s Hospital, University Medical Center Rotterdam, Rotterdam, The Netherlands; 7 Department of Public Health, Erasmus MC, University Medical Center Rotterdam, Rotterdam, The Netherlands

## Abstract

**Background:**

Neonatal hyperbilirubinemia is a leading cause of hospitalization during the first week of life. Recent research suggest that phototherapy, the standard treatment, can be safely and effectively administered at home. Some Dutch hospitals have already adopted home-based phototherapy. The TREAT Jaundice@home study aims to contribute to its broader implementation across the Netherlands. Understanding the perspectives, perceptions, and needs of healthcare professionals is essential for facilitating this implementation.

**Methods:**

This cross-sectional survey targeted pediatricians, midwives, and maternity care assistants with and without prior experience with phototherapy at home. The 82-item questionnaire covered respondent background, experience, interests, motivation, responsibilities, logistics, collaboration, knowledge, indications and contraindications, financial aspects, and implementation readiness.

**Results:**

The study included responses from 16 pediatricians, 90 community midwives, and 514 maternity care assistants. Findings indicate a positive reception of phototherapy at home, regardless of prior experience. The majority expressed satisfaction, recognized potential benefits, and/or demonstrated a willingness to adopt this innovation. Key challenges identified include the need for information, the lack of guidelines, coordination and collaboration issues, and concerns about financial compensation.

**Discussion:**

Phototherapy at home is well-received by healthcare professionals. Addressing the identified challenges is imperative for successful implementation, ultimately benefiting neonates, their families, and healthcare systems.

**Impact:**

- Phototherapy at home is well-received and perceived as beneficial by healthcare professionals with and without prior experience
- Key challenges include the need for better knowledge and guidelines, coordination and collaboration issues among healthcare professionals, and concerns about financial compensation
- Addressing these challenges through comprehensive information, standardized protocols, improved collaboration, and adequate financial compensation is essential to successfully implement phototherapy at home on a larger scale

## Introduction

Neonatal jaundice is a common condition in the first days of life, affecting more than 80% of full-term neonates.^1^ It is caused by a temporary rise in serum bilirubin levels and it is usually a self-limiting condition.^2^ However, severe neonatal hyperbilirubinemia can lead to acute or chronic bilirubin encephalopathy or even result in death. The number of full-term neonates developing clinically significant jaundice, defined as hyperbilirubinemia requiring close monitoring and treatment with phototherapy (PT), varies from 4% to 8%.^1,3–5^ PT is a safe and effective treatment to prevent the bilirubin from reaching hazardous levels by inducing structural and configurational modifications in the bilirubin molecule, which can then be more easily excreted.^6,7^ Currently, PT is typically applied in hospitals. Neonatal hyperbilirubinemia is a major cause for hospitalization in the first week of life, accounting for up to 35% of hospital (re)admission.^3^

A recent systematic review indicates that PT can be applied at home safely and effectively for uncomplicated pathological and physiological neonatal hyperbilirubinemia.^8^ PT at home can also contribute to better patient outcomes by lowering parental stress and anxiety, reducing mother-infant separation, and promoting skin-to-skin contact and breastfeeding.^9–12^ Furthermore, a recent study suggests that a key advantage of PT at home is the empowerment of parents and promotion of parental autonomy.^13^ Finally, PT at home can reduce hospital admissions and associated costs.^14^

In the Netherlands, a few hospitals already facilitate PT at home. Nevertheless, healthcare professionals’ experiences, perceptions, and needs regarding PT at home have not yet been investigated systematically. In addition, the perspectives of healthcare professionals without prior experience with PT at home have only been explored in a limited number of smaller studies from the United States that have been conducted a couple of decades ago.^15–17^ To facilitate the broader implementation of PT at home, it is crucial to understand healthcare professionals’ interests, perceptions, and needs regarding this innovation. This study aims to investigate these perspectives among professionals with or without prior experience with PT at home.

## Materials and methods

### Study design

This cross-sectional survey study administered an online questionnaire to healthcare professionals (HCPs) to evaluate their experiences, interests, perceptions and needs concerning PT at home. This study is part of the TowaRds implEmentATion of phototherapy for neonatal jaundice at home (TREAT Jaundice @ home) study. The overarching aim of TREAT Jaundice @ home is to contribute to the broader implementation of PT at home in the Netherlands. This study is reported according to the consensus-based checklist for reporting of survey studies (CROSS checklist)^18^ and the Revised Standards for Quality Improvement Reporting Excellence (SQUIRE 2.0).^19^

### Population

This survey targeted all HCPs from two regions in the Netherlands involved in conducting PT at home, namely pediatricians from non-tertiary hospitals, community midwives, and maternity care assistants (MCAs). In the Netherlands, the involvement of these HCPs in implementing PT at home is essential due to the unique organization of maternity care, differing from practices in many other countries. Women at low risk of complications may choose to give birth at home, in a primary care birth center, or in a hospital. After giving birth, women and their newborns are usually supported at home by community midwives and MCAs.

### Questionnaire development

The development of the questionnaire followed the procedure outlined in the book “Undertaking Midwifery Research”^20^ and aligns with the questionnaire development process of several other survey studies.^21–23^ The aims and framework of the survey study were discussed within the project group. Subsequently, survey domains were determined by conducting a literature review and by conducting a series of five focus group interviews with a total of 24 HCPs who participate in the TREAT Jaundice @ home study. In addition, elements from existing questionnaires related to PT at home^15^ and healthcare innovation implementation^24–26^ were incorporated. The questionnaire underwent multiple refinement stages, including feedback from the project team, feedback from professionals unfamiliar with the topic, and a pilot study. The pilot study involved two pediatricians, two community midwives, and two MCAs, who represented the study sample. After the pilot, small changes were made to improve clarity and readability of questions.

The final questionnaire comprised 82 questions covering eight domains: (1) background information of the respondents, (2) experience with PT at home, (3) interests and motivation, (4) responsibility, (5) logistics and collaboration, (6) knowledge and information provision, (7) indications and contraindications, and (8) financial aspects and implementation readiness (Supplemental information 1). Since the questionnaire was administered to various HCPs with and without prior experience with PT at home, ‘routing’ was used.^27^ This means, depending on respondents’ occupation and experience with PT at home, they were presented with questions appropriate to their situation.

### Questionnaire administration

LimeSurvey (version 3.28.73) was used to construct the questionnaire. Multistage sampling was employed to reach community midwives and pediatricians. On March 28th, 2023, we reached out to all hospital departments of pediatrics/neonatology and community midwifery practices within the Southwest and Northern Netherlands. Our focus on HCPs from these regions was deliberate, as they were actively participating in the overarching TREAT Jaundice @ home study.

We sent one email with a link to the questionnaire to each community midwifery practice and pediatric department and asked whether one HCP working in that organization would be willing to complete the questionnaire. To reduce the likelihood that the questionnaire was completed by the person with the most affinity for the topic, we asked the institution whether the questionnaire could be completed by the HCP whose birthday was coming up next. We sent a maximum of three reminder emails to organizations that had not completed the questionnaire and closed the questionnaire on June 1st, 2023. Multiple participation was not possible since all organizations received a link that could be used only once.

For approaching MCAs we used a different approach due to the presence of a few large maternity care agencies and numerous smaller organizations and self-employed individuals working within the two regions. Therefore, we used non-probability sampling by distributing a national open-access online questionnaire through the Knowledge Center for Maternity Care (Kenniscentrum Kraamzorg, KCKZ; www.kckz.nl). This knowledge center shared the weblink to the open access questionnaire in their newsletter, social media, and on their website on March 28th 2023 and on May 16th 2023, reaching approximately 7000 MCAs in the Netherlands. The open-access questionnaire was closed on June 19th 2023. A similar strategy was employed in a recent study on MCAs’ knowledge of hyperbilirubinemia.^28^ We screened for multiple participation by examining responses with identical demographic characteristics. If multiple participation was identified, we retained the response with a higher number of completed questions, and in instances where participants completed the questionnaire more than once, we preserved the initial response. Participation in the survey was entirely voluntary; no financial or other incentives were offered.

### Questionnaire completion and data analysis

Questionnaires were included if respondents completed the section on demographic characteristics and at least one other section. For those with prior experience with PT at home this was the section ‘experience’ and for those without prior experience with PT at home this was the section ‘interest and motivation’. In case respondents discontinued the questionnaire after these sections, their responses were included up to the point of discontinuation.

Descriptive analyses were conducted on the data. Numbers and percentages were reported for nominal and ordinal variables. For continuous data, we presented the mean and standard deviation (SD) for normally distributed variables and the median and interquartile range (IQR) for non-normally distributed variables. Considering the expected small sample size of pediatricians and community midwives, their results were combined in the description. For the same reason, no statistical tests were conducted to assess differences between HCPs.

Responses to an open-ended question addressing prerequisites for implementing PT at home were analyzed qualitatively by assigning one or more open code(s) to each response. Subsequently, the coded responses were grouped into distinct code groups (axial codes) and overarching themes were identified.

### Ethical considerations

The study was approved by the ethics committees of the University of Applied Sciences in Rotterdam (reference number 20230201). All respondents provided written informed consent. Data were anonymized in compliance with the Dutch Protection Act for Personal Data. Secure data storage measures were in place to safeguard respondents confidentiality and data integrity.

## Results

### Characteristics of the respondents

The questionnaire was distributed among 18 hospitals and 132 community midwifery practices, yielding responses from 114 individuals. A total of eight respondents discontinued participation after completing the initial section on background information and were consequently excluded. This led to the inclusion of 106 individuals in the study, resulting in a response rate of 71%. A total of six respondents partially completed the questionnaire, and their responses were included up to the point of discontinuation. Among the respondents, there were 16 pediatricians and 90 community midwives. The median age of the respondents was 40 years (Interquartile range (IQR) 35–48), with 98% identifying themselves as female. Median work experience was 14 years (IQR 8–18). The majority (*n* = 71, 67%) of the respondents worked in the Southwest region of the Netherlands.

The questionnaire for MCAs yielded responses from 693 individuals. A total of 179 respondents discontinued participation after completing the initial section on background information or did not complete the informed consent form, and were consequently excluded. This led to the inclusion of 514 out of the potential 7000 MCAs (response rate 7%). A total of 111 individuals partially completed the questionnaire, and their responses were included up to the point of discontinuation. There was no multiple participation identified. MCAs had a median age of 54 years (IQR 19–77), with 99.5% identifying themselves as female. Median work experience was 20 years (IQR 6–30), and representation was noted from all twelve provinces in the Netherlands.

### Experience with PT at home

**Fig. 1:**
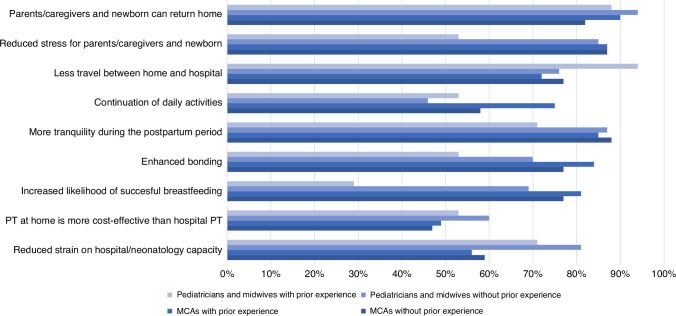
Potential benefits of phototherapy at home. Multiple answers were possible; MCA maternity care assistant, PT phototherapy.

A total of 85 HCPs (14%; 4 pediatricians, 13 community midwives and 68 MCAs) reported having prior experience with PT at home, and subsequently completed the section on ‘Experience with PT at home’. The duration of this experience varied, with about one third having less than one year of experience. HCPs indicated to have experience with either the BiliCocoon Bag (NeoMedLight, Villeurbanne, France) or the Bilisoft blanket (GE Healthcare, Chicago). Respondents highlighted several benefits of PT at home (Fig. 1). Among pediatricians and community midwives, the most commonly cited benefits (mentioned by >70%) included reduced travel between home and hospital for parents and newborn (*n* = 16, 94%), the ability for parents/caregivers and newborns to return home (*n* = 15, 88%), more tranquility during the postpartum period (*n* = 12, 71%), and decreased strain on hospital capacity (*n* = 12, 71%). For MCAs, the primary benefits (mentioned by >70%) were identified as reduced travel between home and hospital for parents and newborn (*n* = 49, 72%), the ability for parents/caregivers and newborns to return home (*n* = 61, 90%), more tranquility (*n* = 58, 85%) and less stress (*n* = 59, 87%) during the postpartum period for parents/caregivers and newborn, continuing daily activity for parents/caregivers (*n* = 51, 75%), improved bonding (*n* = 57, 84%) and increased likelihood of successful breastfeeding (*n* = 55, 81%).

**Fig. 2:**
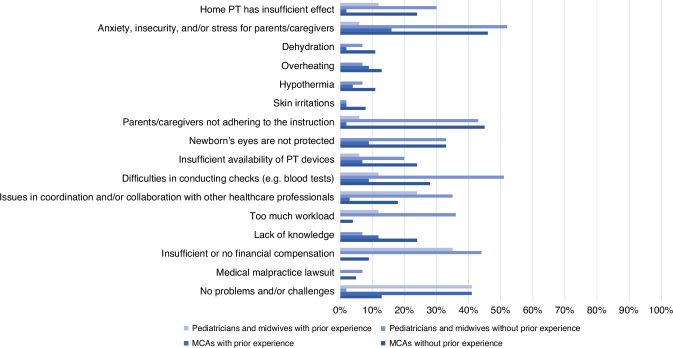
Potential problems or challenges of phototherapy at home. Multiple answers were possible, MCA maternity care assistant, PT phototherapy.

The 85 respondents with experience were queried about possible problems or challenges they encountered with PT at home. 41% of respondents with experience reported no problems or challenges (Fig. 2). Pediatricians and community midwives who encountered issues mentioned difficulties in coordination and collaboration with other HCPs (*n* = 4, 24%) and insufficient or absent financial compensation (*n* = 6, 35%). All six respondents who identified financial compensation as a potential challenge were community midwives. Among MCAs, most frequently mentioned challenges included anxiety, uncertainty and stress for parents/caregivers (*n* = 11, 16%) and a lack of knowledge (*n* = 8, 12%).

The majority of 85 respondents with experience expressed satisfaction with the implementation of PT at home (pediatricians and community midwives: *n* = 12, 71%; MCAs: *n* = 46, 69%). Most participants believed that newborns should have the possibility to receive treatment at home, deeming it both feasible and valuable for their respective organizations (Supplemental information 2).

### Interest and motivation

The section addressing interest and motivation involved the participation of 89 (84%) pediatricians and community midwives and 449 (87%) MCAs lacking prior experience with PT at home. Pediatricians, community midwives and MCAs identified similar advantages associated with PT at home compared to those with prior experience (Fig. 1). However, it is noteworthy that most respondents without prior experience foresee problems or challenges, which were not generally perceived by those with experience. Potential challenges mentioned by more than 40% of pediatricians and community midwives were anxiety, insecurity or stress experienced by parents (*n* = 46, 52%), parents not adhering to scheduled appointments (*n* = 38, 43%), problems with executing controls (*n* = 45, 51%) and concerns related to insufficient or a lack of financial compensation (*n* = 39, 44%). The latter concern was predominately expressed by community midwives. Problems or challenges mentioned by more than 40% of MCAs were anxiety, insecurity or stress experienced by parents (*n* = 206, 46%) and parents not adhering to scheduled appointments (*n* = 201, 45%). A total of 2 (2%) pediatricians and midwives and 56 (13%) MCAs expected no problems or challenges.

The majority of 538 respondents without experience expressed agreement with the notion that newborns with hyperbilirubinemia should have the possibility of receiving treatment at home (pediatricians and community midwives: *n* = 71, 80%; MCAs: *n* = 350, 78%). Additionally, most respondents concurred that the introduction of PT at home is both feasible and worthwhile for their respective healthcare organizations (Supplemental information 2).

### Responsibility, logistics and collaboration

All respondents were queried about topics related to responsibility, logistics and collaboration, resulting in responses from 106 pediatricians and community midwives and 501 MCAs (13 MCAs opted out of the questionnaire at the start of this section). Most respondents suggested that the pediatrician should hold responsibility for PT at home (pediatricians and community midwives: *n* = 82, 77%, MCAs: *n* = 281, 56%), along with the community midwife (pediatricians and community midwives: *n* = 59, 56%; MCA *n* = 373, 75%). Regarding blood sampling, half of the respondents identified the community midwife as the responsible party (pediatricians and community midwives: *n* = 54, 51%; MCA: *n* = 269, 54%), while others mentioned the pediatrician (pediatricians and community midwives: *n* = 29, 27%; MCA: *n* = 82, 16%) or the laboratory (pediatricians and community midwives: *n* = 23, 22%; MCA: *n* = 150, 30%).

The majority of respondents indicated that community midwives should be routinely consulted in PT at home care decisions (pediatricians and community midwives: *n* = 91, 86%; MCA: *n* = 416, 88%). This consideration was based on factors such as midwives’ understanding of families and practical feasibility of the intervention. In addition, many respondents indicated that MCAs should routinely participate (pediatricians and community midwives: *n* = 40, 38%; MCA: *n* = 267, 57%) or at least be informed about this decision (pediatricians and community midwives: *n* = 85, 80%; MCA: *n* = 404, 86%). This was motivated by MCAs’ knowledge of families and the need for organizations to ensure the assignment of experienced MCAs. Nevertheless, only half of pediatricians and community midwives who had prior experience with PT at home reported actual consultations between pediatricians and midwives, and only a minority reported actual consultation with MCAs. Moreover, MCAs also were generally not informed about the decision to treat a newborn at home.

### Knowledge and information provision

All respondents where queried about topics related to knowledge and information provision, resulting in responses from 102 pediatricians and community midwives and 464 MCAs (4 pediatricians and community midwives and 37 MCAs opted out of the questionnaire before this section). Both HCPs with and without prior experience with PT at home expressed a need for additional information regarding PT at home. Most respondents indicated a desire for more information on how the PT device operates (pediatricians and community midwives *n* = 89, 88%; MCAs *n* = 425, 91%) and the required assessments of the newborn (pediatricians and community midwives *n* = 82, 80%; MCAs *n* = 422, 91%). Respondents without prior experience with PT at home also expressed a need for information regarding the benefits of PT at home and its logistical aspects. Pediatricians and community midwives, regardless of their experience, expressed a need for information on financial compensation (Supplemental information 3). Pediatricians and community midwives indicated a preference for online meetings or e-learnings, while MCAs favored in-person meetings in combination with e-learnings (Supplemental information 3).

### Indications and contraindications

The section on indications and contraindications was exclusively presented to pediatricians. A total of 15 pediatricians, both with and without prior experience, completed this section. There was no consensus with respect to the gestational age and age at which treatment at home should be offered. Contra-indications for PT at home that were mentioned by at least half of the respondent include a pathological cause for hyperbilirubinemia (*n* = 13, 87%), language barrier of the parents (*n* = 11, 73%), parental difficulty in understanding the treatment (*n* = 15, 100%), intellectual disability of parents (*n* = 9, 60%), parental preference for hospital care (*n* = 10, 67%) and hesitation expressed by midwives or doctors (*n* = 13, 87%; Supplemental information 4).

### Financial aspects and implementation readiness

All respondents where queried about topics related to financial aspects and implementation, resulting in responses from 100 pediatricians and community midwives and 403 MCAs (2 pediatricians and community midwives and 61 MCAs opted out of the questionnaire before this section). Among the 15 pediatricians and community midwives with prior experience, eight stated that they do not receive any financial compensation for PT at home. Five respondents expressed uncertainty about the compensation, while two mentioned receiving compensation that falls short of covering all costs. Nonetheless, the majority of community midwives with experience (*n* = 10, 83%) and one pediatrician (*n* = 1, 33%) acknowledged that PT at home demands additional time. Pediatricians and community midwives without experience anticipated that PT at home would require additional time (*n* = 70, 82%) and indicated that it necessitates financial compensation (*n* = 74, 87%).

**Fig. 3:**
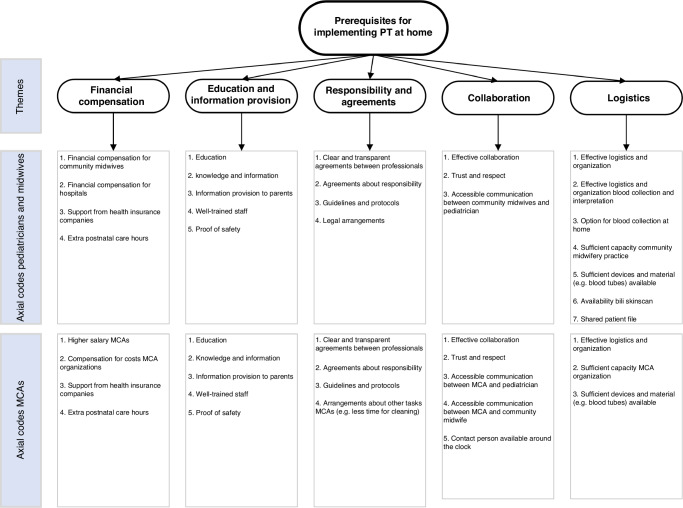
Qualitative analyses of questions about requirements and needs among pediatricians and community midwives. PT phototherapy, MCA maternity care assistant.

In total, 66 pediatricians and community midwives and 211 MCAs with and without prior experience with PT at home responded to the open-ended question about prerequisites for implementing PT at home. Each response generated one or more open codes, resulting in a total of 236 codes for pediatricians and community midwives and 490 codes for MCAs. Based on the analyses, five core themes were established: (1) financial compensation, (2) education and information provision, (3) responsibility and clear agreements, (4) collaboration, and (5) logistics. Respondents expressed a need for information and training about PT at home. In addition, financial compensation emerged as an important theme with the majority of pediatricians and community midwives mentioning that the implementation of PT at home requires financial compensation for community midwives. Among the MCAs, financial compensation also emerged as an important theme with many MCAs mentioning that the increase in responsibility would require a higher salary for MCAs (Fig. 3).

Respondents were surveyed about their readiness for implementing PT at home using the Organizational readiness for implementing change (ORIC) questionnaire. The majority indicated agreement or partial agreement with all statements on implementation readiness (Supplemental information 5).

## Discussion

This study explored the perspectives of HCPs, including pediatricians, community midwives, and MCAs, regarding PT at home in the Netherlands. The results revealed that PT at home is generally well-received by HCPs with or without having prior experience with PT at home. The majority of respondents expressed satisfaction with its implementation and/or recognized its potential benefits and demonstrated willingness to adopt this innovation. However, some challenges were identified. These included the need for knowledge and information provision, the lack of guidelines and protocols, coordination and collaboration issues among HCPs, and concerns about financial compensation.

Strengths of this study include the comprehensive questionnaire development process and the inclusion of different groups of stakeholders, including pediatricians, community midwives as well as MCAs with and without prior experience with PT at home. This has led to a comprehensive assessment of relevant perspectives of HCPs on PT at home. In addition, the response rate for pediatricians and community midwives was high (71%).^29^

Limitations include the relatively low response rate for MCAs (7%). This may cause non-response bias, which is a common challenge in healthcare professionals’ surveys.^30,31^ In addition, although the response rate among pediatricians was relatively high (we received responses from 16 out of 18 hospitals; 89%), the absolute number of pediatricians in our study (*n* = 16) was low. Consequently, we reported the results for pediatricians and community midwives together and we refrained from undertaking a comparative analysis between HCPs.

Implementation research has demonstrated that the success of an implementation is closely linked to stakeholders’ perceptions of its acceptability, appropriateness, and feasibility.^32,33^ In alignment with findings from a recent qualitative study on PT at home for neonatal jaundice from Australia,^13^ participants in our study generally expressed favorable attitudes towards the implementation of PT at home. The majority agreed that newborns should have the possibility to be treated with PT at home, deeming it both feasible and valuable in the treatment of neonatal hyperbilirubinemia. Moreover, HCPs acknowledged multiple benefits associated with PT at home and indicated a sense of readiness for the implementation of PT at home. These factors may positively impact the success of implementing PT at home.

Recent studies on PT at home suggest it is generally safe, which may also explain the positive attitude among HCPs. A recent randomized controlled trial reported a low hospital readmission rate of 3.8% due to unsuccessful PT at home.^34^ Common reasons for readmission in this study and other studies included reduced PT exposure due to language and cultural barriers, non-compliance with treatment protocols, parental anxiety, and clinical concerns such as temperature instability in the newborn.^34–36^ None of the readmitted neonates required exchange transfusion or developed acute bilirubin encephalopathy or kernicterus spectrum disorders. Most studies utilized the BiliCocoon Bag (NeoMedLight, Villeurbanne, France) or the BiliSoft blanket (GE Healthcare, Chicago), which are also used for PT at home in the Netherlands.

Despite the positive attitude of HCPs and favorable results from recent studies, the current investigation also identified several challenges and necessary preparatory steps for the successful implementation of PT at home. Although HCPs without experience with PT at home highlighted numerous potential advantages, often comparable or even surpassing those cited by respondents with experience, they also identified a greater number of potential problems or challenges. Anticipating these issues may influence respondents’ perceptions of acceptability, appropriateness, and feasibility, potentially impeding the successful implementation of PT at home.^32,33^ Therefore, it is crucial to integrate the potential problems or challenges identified by respondents without experience, such as anxiety, insecurity and/or stress for parents/caregivers, non-adherence to instructions by parents/caregivers, and problems with executing controls, into education and training to address these concerns effectively. In addition, respondents expressed a desire for more comprehensive information about how the PT device operates. Tailored education approaches such as online meetings or e-learning modules for pediatricians and midwives, and in-person meetings in combination with e-learnings for MCAs, were suggested to address these needs. The need for education and information was also identified as a prerequisite for implementing PT at home in the recent qualitative study conducted by Anderson et al. in Australia,^13^ and was deemed significant in another survey study focusing on the implementation of innovations in children’s health care.^37^

Another noteworthy finding in this survey pertains to the necessity of clear guidelines and protocols for PT at home. There appears to be a lack of consensus among physicians regarding the indications and contra-indications of PT at home. Additionally, well-defined guidelines and arrangements concerning logistics, collaborations, and responsibilities were mentioned as prerequisites for implementing PT at home. Effective collaboration between pediatricians, community midwives, and MCAs is also crucial, and according to our study, midwives and MCAs should be routinely consulted and informed about decisions related to PT at home care. This emphasis on the importance of establishing clear procedures aligns with the conclusions drawn by Anderson et al.^13^

Ultimately, a crucial factor to take into account is the financial dimension of PT at home. Currently, a lack of financial compensation for community midwives is perceived as a significant obstacle to implement PT at home. Additionally, MCAs indicated that taking on additional responsibilities associated with PT at home should result in higher remuneration for their services. In a survey regarding home-based monitoring and telemonitoring in perinatal care in the Netherlands, HCPs also reported challenges with reimbursements, due to the absence of insurance coverage.^38^ Financial concerns were the primary reason for not offering home-based monitoring and telemonitoring in this study.^38^ This was in line with two other survey studies from Saudi Arabia and Germany about “hospital at home” interventions^39,40^ and underscores the need for fair and transparent compensation models to motivate HCPs to offer PT at home.

## Conclusion

PT at home is generally well-received and perceived as beneficial according to healthcare professionals. Yet, to successfully implement PT at home on a larger scale, addressing the perceived challenges and concerns on adequate support, education, and financial compensation for healthcare professionals are essential. Furthermore, a clear guideline on management of PT at home should be established for a safe and appropriate provision of PT at home, ultimately benefiting both neonates and their families as well as health care systems.

## Supplementary information

Supplementary information

## Data Availability

The dataset generated during and/or analyzed during the current study are available from the corresponding author on reasonable request.
