# Supplementary material for: Home phototherapy for neonatal hyperbilirubinemia: current practices and attitudes
Source: Pediatr Res. 2024 Dec 13;98(2):512–8. doi: 10.1038/s41390-024-03754-8 (PMC12454146; doi:10.1038/s41390-024-03754-8)
Supplement: Supplementary file 1 — Supplementary information [file 41390_2024_3754_MOESM1_ESM.pdf]

S1. Questionnaire

Vragenlijst invoering van fototherapie thuis voor pasgeborenen met geelzucht/hyperbilirubinemie in het kader van de *TREAT Jaundice @ home study*

Toestemming
Ik heb de informatie over het onderzoek gelezen en begrepen.
Ik weet dat deelname vrijwillig is en wat er met mijn gegevens gebeurt.
Ik geef toestemming voor deelname aan dit vragenlijstonderzoek.

- o Ja
- o Nee (u wordt gelijk naar het einde van deze vragenlijst geleid)

Indien Nee → deelnemer moet naar het einde van de vragenlijst worden geleid)

Deel 1: Achtergrondinformatie van de respondenten

Wat is uw leeftijd?
…. Jaar

Wat is uw geslacht?

- o Vrouw
- o Man
- o Anders
- o Wil ik niet zeggen

Wat is uw beroep?

- o Kinderarts of Kinderarts-neonatoloog
- o Physician Assistant of Verpleegkundig Specialist kindergeneeskunde
- o Eerstelijns verloskundige
- o Kraamverzorgende werkende bij mensen thuis of in een geboortecentrum

Hoeveel jaar bent u werkzaam als professional in uw huidige beroep?
Indien u minder dan één jaar werkzaam bent in uw huidige beroep, kunt u '0' invullen.
Ik ben nu …. jaar werkzaam in dit beroep

IF beroep = arts, PA, verloskundige
In welke regio bent u de meeste tijd werkzaam?
In de regio van VSV/ IGO:

- o VSV Dirksland (IGO Zuid aan Zee)
- o VSV Dordrecht e.o.
- o VSV Erasmus MC / Geboortecentrum Sophia
- o VSV IJsselland e.o.
- o VSV Reinier (IGO Reinova)
- o VSV Rotterdam Zuid (Ikazia en Maasstad ziekenhuis)
- o VSV Sint Franciscus Gasthuis
- o VSV Vlietland
- o VSV Breda e.o. (IGO Annature)
- o VSV West-Brabant (IGO Qocon)
- o VSV ADRZ Zeeland
- o VSV Zeeuws-Vlaanderen
- o VSV Sneek
- o VSV Stad en Ommeland
- o VSV Treant
- o VSV Rondom Zwangerschap Noord-Friesland
- o VSV Ommelander

- VSV Middenin
- VSV Martini
- VSV Drachten
- VSV Assen

IF beroep = kraamverzorgende

In welke provincie bent u de meeste tijd werkzaam?

- Groningen
- Friesland
- Drenthe
- Overijssel
- Flevoland
- Gelderland
- Utrecht
- Noord-Holland
- Zuid-Holland
- Zeeland
- Noord-Brabant
- Limburg

IF beroep = Kinderarts, PA

In wat voor type ziekenhuis bent u de meeste tijd werkzaam?

- Academisch ziekenhuis
- Topklinisch ziekenhuis of algemeen ziekenhuis

IF beroep = kraamverzorgende

Waar werkt u als kraamverzorgende? *meerdere antwoorden zijn mogelijk*

- ☐ Bij mensen thuis
- ☐ In een (eerstelijns) geboortecentrum / geboortehotel / geboortekliniek / kraamhotel

Deel 2: Ervaring

Geelzucht/hyperbilirubinemie bij pasgeborenen is een veelvoorkomend fenomeen. Het is één van de belangrijkste oorzaken van opname in het ziekenhuis in de eerste levensweek. Het is echter mogelijk om fototherapie thuis aan te bieden. Ouder(s)/verzorger(s) krijgen dan een apparaat (bijvoorbeeld een slaapzak of matje met blauw licht) mee naar huis. Op deze manier hoeft de pasgeborene niet opgenomen te worden.

Hoe vaak heeft u de afgelopen 12 maanden een pasgeborene met geelzucht/hyperbilirubinemie gezien, die daarvoor behandeling nodig had? Behandeling kan thuis of in het ziekenhuis zijn geweest.

- Nooit
- 1 of 2 keer
- 3 tot 5 keer
- 6 tot 10 keer
- 11 tot 20 keer
- Meer dan 20 keer

Er zijn al een aantal ziekenhuizen in Nederland die fototherapie thuis aanbieden. Heeft u ervaring met de thuisbehandeling van geelzucht/hyperbilirubinemie met fototherapie? U werkt bijvoorbeeld in een ziekenhuis dat (soms) pasgeborenen met geelzucht/hyperbilirubinemie met fototherapie thuis behandelt (als standaard zorg of als onderdeel van een pilot onderzoek). Of u heeft als verloskundigen of kraamverzorgende de zorg over een pasgeborene gehad die thuis of in een geboortecentrum werd behandeld met fototherapie.

- Ja
- Nee

IF beroep = arts, PA & ervaring = ja

Is de thuisbehandeling met fototherapie voor geelzucht/hyperbilirubinemie in uw ziekenhuis onderdeel van een pilotstudie of lopend onderzoek?

- Ja
- Nee

IF beroep = arts, PA & ervaring = ja

Hoe lang heeft uw ziekenhuis al ervaring met de thuisbehandeling van geelzucht/hyperbilirubinemie met fototherapie?

- Minder dan 1 jaar
- 1 of 2 jaar
- 3 of 4 jaar
- 5 jaar of langer

IF beroep = arts, PA & ervaring = ja

Kunt u een schatting maken van hoeveel pasgeborenen uw ziekenhuis per maand thuis of in een geboortecentrum behandelt met fototherapie. *Indien dit minder dan één pasgeborene per maand is, kunt u hier '0' invullen.*

…… pasgeborenen worden er gemiddeld per maand thuis behandeld met fototherapie

Limesurvey: restrictie zetten zodat er alleen getallen kunnen worden ingevuld

IF beroep = verloskundige | kraamverzorgende & ervaring = ja

Hoe lang levert u al zorg voor pasgeborenen die thuis en/of in een geboortecentrum worden behandeld met fototherapie i.v.m. geelzucht/hyperbilirubinemie?

- Minder dan 1 jaar
- 1 of 2 jaar

- ○ 3 of 4 jaar
- ○ 5 jaar of langer

IF beroep = verloskundige | kraamverzorgende & ervaring = ja

Kunt u een schatting geven hoe vaak u de afgelopen 12 maanden de zorg heeft gehad voor een pasgeborene die thuis en/of in een geboortecentrum werd behandeld met fototherapie i.v.m. geelzucht/hyperbilirubinemie?

Ongeveer ….. keer meegemaakt dat ik de zorg had voor een pasgeborene die thuis / in een geboortecentrum werd behandeld met fototherapie

Limesurvey: restrictie zetten zodat er alleen getallen kunnen worden ingevuld

IF ervaring met fototherapie = ja

Met wat voor apparaat/apparaten heeft u de afgelopen 12 maanden gewerkt?

Meerdere antwoorden zijn mogelijk

- ☐ Fototherapie slaapzak (bijvoorbeeld BiliCocoon)
- ☐ Fototherapie matje (bijvoorbeeld Biliblanket/BiliSoft)
- ☐ Fototherapielampen van bovenaf (in de ziekenhuissetting)
- ☐ Anders, namelijk…………………………………………….

IF ervaring met fototherapie = ja

Wat zijn volgens u de voordelen van thuisbehandeling met fototherapie voor geelzucht/hyperbilirubinemie?

Meerdere antwoorden zijn mogelijk

- ☐ Ouder(s)/verzorger(s) en pasgeborene kunnen eerder naar huis en/of hoeven niet opgenomen te worden in het ziekenhuis
- ☐ Minder stress voor ouder(s)/verzorger(s) en/of pasgeborene
- ☐ Minder reizen tussen thuis en ziekenhuis voor ouder(s)/verzorger(s)
- ☐ Voortzetten van dagelijkse activiteiten voor ouder(s)/verzorger(s)
- ☐ Meer rust in de kraamweek
- ☐ Betere hechting tussen ouder(s)/verzorger(s) en pasgeborene
- ☐ Grotere kans op het slagen van borstvoeding
- ☐ Fototherapie thuis is goedkoper dan fototherapie in het ziekenhuis
- ☐ Minder belasting ziekenhuis / neonatologie-capaciteit
- ☐ Ik zie geen voordelen van de thuisbehandeling met fototherapie
- ☐ Anders, namelijk………………….

IF ervaring met fototherapie = ja & bij voordelen is NIET de optie "ik zie geen voordelen" aangevinkt

En welke van de zojuist aangevinkte voordelen is voor u het belangrijkst?

- ○ Ouder(s)/verzorger(s) en pasgeborene kunnen eerder naar huis en/of hoeven niet opgenomen te worden in het ziekenhuis
- ○ Minder stress voor ouder(s)/verzorger(s) en/of pasgeborene
- ○ Minder reizen tussen thuis en ziekenhuis voor ouder(s)/verzorger(s)
- ○ Voortzetten van dagelijkse activiteiten voor ouder(s)/verzorger(s)
- ○ Meer rust in de kraamweek
- ○ Betere hechting tussen ouder(s)/verzorger(s) en pasgeborene
- ○ Grotere kans op het slagen van borstvoeding
- ○ Fototherapie thuis is goedkoper dan fototherapie in het ziekenhuis
- ○ Minder belasting ziekenhuis / neonatologie-capaciteit
- ○ Anders, namelijk……

IF ervaring met fototherapie = ja

Welke problemen of uitdagingen bent u de afgelopen twaalf maanden tegengekomen bij het toepassen van fototherapie thuis voor geelzucht/hyperbilirubinemie?

Meerdere antwoorden zijn mogelijk

- ☐ Fototherapie thuis had te weinig effect, waardoor een ziekenhuisopname alsnog nodig was
- ☐ Angst, onzekerheid en/of stress bij ouder(s)/verzorger(s)
- ☐ Uitdroging bij de pasgeborene
- ☐ Oververhitting bij de pasgeborene
- ☐ Onderkoeling bij de pasgeborene
- ☐ Huidirritaties bij de pasgeborene
- ☐ Ouder(s)/verzorger(s) die zich niet aan de afspraken houden
- ☐ Ouder(s)/verzorger(s) die de ogen van de pasgeborene niet goed beschermen en/of oogbeschermers die niet goed blijven zitten
- ☐ Te weinig apparaten beschikbaar voor fototherapie thuis
- ☐ Problemen met het uitvoeren van controles (o.a. Bloedprikken)
- ☐ Problemen in de afstemming en/of samenwerking met andere zorgprofessionals
- ☐ Het leverde mij teveel werk op
- ☐ Ik wist niet wat ik moest doen / ik kon ouder(s)/verzorger(s) geen goede uitleg geven
- ☐ Een te lage/geen financiële vergoeding
- ☐ Medische tuchtzaak
- ☐ Er waren geen problemen en/of uitdagingen
- ☐ Anders, namelijk……………..

IF ervaring met fototherapie = ja & bij voordelen is NIET de optie "Geen. Ik voorzie geen problemen en/of uitdagingen" aangevinkt

En welke van de zojuist aangevinkte problemen en/of uitdagingen is voor u het belangrijkst?

- o Fototherapie thuis had te weinig effect, waardoor een ziekenhuisopname alsnog nodig was
- o Angst, onzekerheid en/of stress bij ouder(s)/verzorger(s)
- o Uitdroging bij de pasgeborene
- o Oververhitting bij de pasgeborene
- o Onderkoeling bij de pasgeborene
- o Huidirritaties bij de pasgeborene
- o Ouder(s)/verzorger(s) die zich niet aan de afspraken houden
- o Ouder(s)/verzorger(s) die de ogen van de pasgeborene niet goed beschermen en/of oogbeschermers die niet goed blijven zitten
- o Te weinig apparaten beschikbaar voor fototherapie thuis
- o Problemen met het uitvoeren van controles (o.a. Bloedprikken)
- o Problemen in de afstemming en/of samenwerking met andere zorgprofessionals
- o Het leverde mij teveel werk op
- o Ik wist niet wat ik moest doen / ik kon ouder(s)/verzorger(s) geen goede uitleg geven
- o Een te lage/geen financiële vergoeding
- o Medische tuchtzaak
- o Anders, namelijk……………..

IF ervaring met fototherapie = ja

Hoe tevreden bent u op dit moment met de invoering van de thuisbehandeling met fototherapie?

Zeer ontevreden	Ontevreden	Neutraal	Tevreden	Zeer tevreden
☐	☐	☐	☐	☐

IF ervaring met fototherapie = ja

Indien u uw antwoord op bovenstaande vraag (Hoe tevreden bent u) wilt toelichten, kunt u dat hier onder doen.

………

………

IF ervaring met fototherapie = ja

Hieronder staan een aantal stellingen over uw interesse en motivatie rondom thuisbehandeling met fototherapie bij pasgeborenen met geelzucht/hyperbilirubinemie. Zou u per stelling willen aangeven in welke mate u het eens bent met de stelling?

IF ervaring met fototherapie = ja

Ik vind dat pasgeborenen met geelzucht/hyperbilirubinemie thuis behandeld zouden moeten kunnen worden met fototherapie

Sterk mee oneens	Mee oneens	Neutraal	Mee eens	Sterk mee eens
☐	☐	☐	☐	☐

IF ervaring met fototherapie = ja

Ik denk dat mijn collega's (ook) vinden dat pasgeborenen met geelzucht/hyperbilirubinemie thuis behandeld zouden moeten worden kunnen worden met fototherapie

Sterk mee oneens	Mee oneens	Neutraal	Mee eens	Sterk mee eens
☐	☐	☐	☐	☐

IF ervaring met fototherapie = ja

Ik denk dat het haalbaar is om (een deel van de) pasgeborenen met geelzucht/hyperbilirubinemie thuis te behandelen met fototherapie

Sterk mee oneens	Mee oneens	Neutraal	Mee eens	Sterk mee eens
☐	☐	☐	☐	☐

IF ervaring met fototherapie = ja

Ik denk dat het zinvol is voor mijn organisatie om pasgeborenen met geelzucht/hyperbilirubinemie thuis te behandelen met fototherapie

Sterk mee oneens	Mee oneens	Neutraal	Mee eens	Sterk mee eens
☐	☐	☐	☐	☐

IF ervaring met fototherapie = ja

Heeft u verbeterpunten met betrekking tot de invoering van thuisbehandeling met fototherapie? Zijn er zaken die anders/beter kunnen?

………

………

………

IF ervaring met fototherapie = nee

Deel 3: Interesses en motivatie

Geelzucht/hyperbilirubinemie bij pasgeborenen is een veelvoorkomend fenomeen en een van de belangrijkste oorzaken van (her)opname in het ziekenhuis in de eerste levensweek. Het is echter mogelijk om fototherapie thuis aan te bieden door ouder(s)/verzorger(s) een apparaat (bijvoorbeeld een slaapzak of matje met blauw licht) mee te geven naar huis. Op deze manier hoeft de pasgeborene niet opgenomen te worden. In het kader van ziekenhuis verplaatste zorg wordt fototherapie thuis al door een aantal ziekenhuizen in Nederland toegepast. In de toekomst wordt dit mogelijk ook aangeboden in uw regio. Wij zijn daarom benieuwd naar uw interesse en motivatie rondom thuisbehandeling met fototherapie bij pasgeborenen met geelzucht/hyperbilirubinemie.

IF ervaring met fototherapie = nee

Hieronder staan een aantal stellingen over uw interesse en motivatie rondom thuisbehandeling met fototherapie bij pasgeborenen met geelzucht/hyperbilirubinemie. Zou u per stelling willen aangeven in welke mate u het eens bent met de stelling?

Ik vind dat pasgeborenen met geelzucht/hyperbilirubinemie thuis behandeld zouden moeten kunnen worden met fototherapie

Sterk mee oneens	Mee oneens	Neutraal	Mee eens	Sterk mee eens
☐	☐	☐	☐	☐

Ik denk dat mijn collega's (ook) vinden dat pasgeborenen met geelzucht/hyperbilirubinemie thuis behandeld zouden moeten worden kunnen worden met fototherapie

Sterk mee oneens	Mee oneens	Neutraal	Mee eens	Sterk mee eens
☐	☐	☐	☐	☐

Ik denk dat het haalbaar is om (een deel van de) pasgeborenen met geelzucht/hyperbilirubinemie thuis te behandelen met fototherapie

Sterk mee oneens	Mee oneens	Neutraal	Mee eens	Sterk mee eens
☐	☐	☐	☐	☐

Ik denk dat het zinvol is voor mijn organisatie om pasgeborenen met geelzucht/hyperbilirubinemie thuis te behandelen met fototherapie

Sterk mee oneens	Mee oneens	Neutraal	Mee eens	Sterk mee eens
☐	☐	☐	☐	☐

IF ervaring met fototherapie = nee

Wat kunnen volgens u voordelen zijn van fototherapie thuis voor pasgeborenen met geelzucht/hyperbilirubinemie?

Meerdere antwoorden zijn mogelijk

- ☐ Ouder(s)/verzorger(s) en pasgeborene kunnen eerder naar huis en/of hoeven niet opgenomen te worden in het ziekenhuis
- ☐ Minder stress voor ouder(s)/verzorger(s) en/of pasgeborenen
- ☐ Minder reizen tussen thuis en ziekenhuis voor ouder(s)/verzorger(s)
- ☐ Voortzetten van dagelijkse activiteiten voor ouder(s)/verzorger(s)
- ☐ Meer rust in de kraamweek
- ☐ Betere hechting tussen ouder(s)/verzorger(s) en kind
- ☐ Grotere kans op het slagen van borstvoeding
- ☐ Fototherapie thuis is goedkoper dan fototherapie in het ziekenhuis

- ☐ Minder belasting ziekenhuis / neonatologie-capaciteit
- ☐ Ik zie geen voordelen van fototherapie thuis
- ☐ Anders, namelijk………………….

IF ervaring met fototherapie = nee & bij voordelen is NIET de optie “ik zie geen voordelen” aangevinkt

En welke van de zojuist aangevinkte voordelen is voor u het belangrijkst?

- o Ouder(s)/verzorger(s) en pasgeborene kunnen eerder naar huis en/of hoeven niet opgenomen te worden in het ziekenhuis
- o Minder stress voor ouder(s)/verzorger(s) en/of pasgeborene
- o Minder reizen tussen thuis en ziekenhuis voor ouder(s)/verzorger(s)
- o Voortzetten van dagelijkse activiteiten voor ouder(s)/verzorger(s)
- o Meer rust in de kraamweek
- o Betere hechting tussen ouder(s)/verzorger(s) en pasgeborene
- o Grotere kans op het slagen van borstvoeding
- o Fototherapie thuis is goedkoper dan fototherapie in het ziekenhuis
- o Minder belasting ziekenhuis / neonatologie-capaciteit
- o Anders, namelijk………………….

IF ervaring met fototherapie = nee

Welke mogelijke problemen en/of uitdagingen voorziet u omtrent het aanbieden van fototherapie thuis?
Meerdere antwoorden zijn mogelijk

- ☐ Dat fototherapie thuis te weinig effect heeft, waardoor een ziekenhuisopname alsnog nodig is
- ☐ Angst, onzekerheid en/of stress bij ouder(s)/verzorger(s)
- ☐ Uitdroging bij de pasgeborene
- ☐ Oververhitting bij de pasgeborene
- ☐ Onderkoeling bij de pasgeborene
- ☐ Huidirritaties bij de pasgeborene
- ☐ Ouder(s)/verzorger(s) die zich niet aan de afspraken houden
- ☐ Ouder(s)/verzorger(s) die de ogen van de pasgeborene niet goed beschermen en/of oogbeschermers die niet goed blijven zitten
- ☐ Te weinig apparaten beschikbaar voor fototherapie thuis
- ☐ Problemen met het uitvoeren van controles (o. a. Bloedprikken)
- ☐ Problemen in de afstemming en/of samenwerking met andere zorgprofessionals
- ☐ Dat het mij teveel werk oplevert
- ☐ Dat ik niet weet wat ik moet doen / ouder(s)/verzorger(s) geen goede uitleg kan geven
- ☐ Een te lage/geen financiële vergoeding
- ☐ Medische tuchtzaak
- ☐ Geen. Ik voorzie geen problemen en/of uitdagingen
- ☐ Anders, namelijk………………

IF ervaring met fototherapie = nee & bij voordelen is NIET de optie “Geen. Ik voorzie geen problemen en/of uitdagingen” aangevinkt

En welke van de zojuist aangevinkte problemen en/of uitdagingen is voor u het belangrijkst?

- o Dat fototherapie thuis te weinig effect heeft, waardoor een ziekenhuisopname alsnog nodig is
- o Angst, onzekerheid en/of stress bij ouder(s)/verzorger(s)
- o Uitdroging bij de pasgeborene
- o Oververhitting bij de pasgeborene
- o Onderkoeling bij de pasgeborene
- o Huidirritaties bij de pasgeborene

- Ouder(s)/verzorger(s) die zich niet aan de afspraken houden
- Ouder(s)/verzorger(s) die de ogen van de pasgeborene niet goed beschermen en/of oogbeschermers die niet goed blijven zitten
- Te weinig apparaten beschikbaar voor fototherapie thuis
- Problemen met het uitvoeren van controles (o.a. Bloedprikken)
- Problemen in de afstemming en/of samenwerking met andere zorgprofessionals
- Dat het mij teveel werk oplevert
- Dat ik niet weet wat ik moet doen / ouder(s)/verzorger(s) geen goede uitleg kan geven
- Een te lage/geen financiële vergoeding
- Medische tuchtzaak
- Geen. Ik voorzie geen problemen en/of uitdagingen
- Anders, namelijk………………

Deel 4: verantwoordelijkheid en logistiek

Als fototherapie thuis wordt aangeboden kan dit op verschillende manieren. Sommige ziekenhuizen in Nederland en wereldwijd hebben het zo geregeld dat zij de volledige zorg dragen voor de pasgeborenen die thuis worden behandeld. Andere ziekenhuizen werken hierin juist samen met eerstelijns verloskundigen en kraamverzorgenden. Wij zijn benieuwd wat uw ideeën hier over zijn.

Wie zou er of zouden er volgens u verantwoordelijk moeten zijn voor de zorg van een pasgeborene die thuis wordt behandeld met fototherapie? Het gaat hierbij om uw mening.
Meerdere antwoorden zijn mogelijk

- ☐ De kinderarts of kinderarts-neonatoloog
- ☐ De eerstelijns verloskundige
- ☐ De kraamverzorgende
- ☐ De ouder(s)/verzorger(s)
- ☐ Anders, namelijk………………………………………

IF ervaring met fototherapie = ja
Wie is of zijn er volgens uw protocol en/of werkafspraken verantwoordelijk voor een pasgeborene die thuis wordt behandeld met fototherapie?
Meerdere antwoorden zijn mogelijk

- ☐ De kinderarts of kinderarts-neonatoloog
- ☐ De eerstelijns verloskundige
- ☐ De kraamverzorgende
- ☐ De ouder(s)/verzorger(s)
- ☐ Anders, namelijk………………………………………

Wie zou er volgens u verantwoordelijk moeten zijn dat er bloed wordt afgenomen bij de pasgeborene die thuis wordt behandeld met fototherapie?

- o De kinderarts / kinderarts-neonatoloog
- o De eerstelijns verloskundige
- o Het huisartsenlaboratorium en/of eerstelijns laboratorium

IF ervaring met fototherapie = ja
Wie is er volgens uw protocol en/of werkafspraken verantwoordelijk voor het afnemen van bloed bij de pasgeborene?

- o De kinderarts / kinderarts-neonatoloog
- o De eerstelijns verloskundige
- o Het huisartsenlaboratorium en/of eerstelijns laboratorium

IF ervaring met fototherapie = ja & de kinderarts is verantwoordelijk
Hoe wordt het bloedprikken logistiek geregeld?

- o De verpleegkundige en/of het lab komt thuis bloedprikken en regelt het transport van het bloed naar het ziekenhuis
- o De verpleegkundige en/of het lab komt thuis bloedprikken en ouder(s)/verzorger(s) regelen het transport van het bloed naar het ziekenhuis
- o Ouder(s)/verzorger(s) gaan met de pasgeborene naar het ziekenhuis om bloed te laten prikken
- o Anders, namelijk……………………………………………………

IF ervaring met fototherapie = ja & de verloskundige is verantwoordelijk

Hoe wordt het bloedprikken logistiek geregeld?

- De verloskundige komt thuis bloedprikken en regelt het transport van het bloed naar het ziekenhuis
- De verloskundige komt thuis bloedprikken en ouder(s)/verzorger(s) regelen het transport van het bloed naar het ziekenhuis
- Anders, namelijk…………………………………………………………

Deel 5: Samenwerking

Wie zouden er volgens uw mening moeten samenwerken bij de zorg voor een pasgeborene die thuis wordt behandeld met fototherapie?
Meerdere antwoorden zijn mogelijk

- ☐ Kinderarts, kinderarts-neonatoloog en/of arts-assistent kindergeneeskunde
- ☐ Physician assistant of verpleegkundig specialist kindergeneeskunde
- ☐ Neonatologie verpleegkundige
- ☐ Eerstelijns verloskundige
- ☐ Het eerstelijns laboratorium
- ☐ Het ziekenhuislaboratorium
- ☐ Kraamverzorgende
- ☐ Ouder(s)/verzorger(s)
- ☐ Sociale netwerk (bijvoorbeeld familieleden) van ouder(s)/verzorger(s)
- ☐ Anders, namelijk…………….

IF ervaring met fototherapie = ja & beroep = arts | verloskundige | PA
Vindt er overleg plaats tussen de afdeling kindergeneeskunde en de eerstelijns verloskundige over welke pasgeborenen in aanmerking komen voor fototherapie thuis?

- o Ja, (bijna) altijd
- o Soms
- o Nee, (bijna) nooit

Zouden eerstelijns verloskundigen volgens uw mening door de afdeling kindergeneeskunde betrokken moeten worden bij de beslissing om fototherapie thuis aan te bieden aan een pasgeborene?

- o Ja, (bijna) altijd
- o Soms
- o Nee, (bijna) nooit

Indien u uw antwoord op bovenstaande vraag (zouden eerstelijns verloskundigen betrokken moeten worden bij de beslissing om fototherapie thuis aan te bieden) wilt toelichten, kunt u dat hier onder doen.
………
………

IF ervaring met fototherapie = ja
Vindt er overleg plaats tussen de afdeling kindergeneeskunde en de betrokken kraamverzorgende over welke pasgeborenen in aanmerking komen voor fototherapie thuis?

- o Ja, (bijna) altijd
- o Soms
- o Nee, (bijna) nooit

IF ervaring met fototherapie = ja
Wordt de kraamverzorgende op de hoogte gesteld door de afdeling kindergeneeskunde als een pasgeborene thuis behandeld gaat worden met fototherapie?

- o Ja, (bijna) altijd
- o Soms
- o Nee, (bijna) nooit

Zouden kraamverzorgenden volgens uw mening door de afdeling kindergeneeskunde betrokken moeten worden bij de beslissing om fototherapie thuis aan te bieden aan een pasgeborene?

- o Ja, altijd
- o Soms
- o Nee, nooit

Zouden kraamverzorgenden volgens uw mening op de hoogte moeten worden gesteld door de afdeling kindergeneeskunde als een pasgeborene thuis behandeld gaat worden met fototherapie?

- o Ja, (bijna) altijd
- o Soms
- o Nee, (bijna) nooit

Indien u uw antwoord op bovenstaande twee vragen (zouden kraamverzorgende betrokken moeten worden bij de beslissing om fototherapie thuis aan te bieden en/of zouden kraamverzorgenden hiervan op de hoogte worden gesteld) wilt toelichten, kunt u dat hier onder doen

………
………

IF beroep = arts, PA

Welke rol(len) zouden eerstelijns verloskundigen volgens uw mening moeten hebben bij pasgeborenen die (in de toekomst) thuis behandeld worden met fototherapie? *Meerdere antwoorden zijn mogelijk*

- ☐ Geen. Eerstelijns verloskundigen zouden hier geen rol in moeten hebben
- ☐ Bloedafname
- ☐ De uitvoering controleren
- ☐ Eerste aanspreekpunt/contactpersoon bij problemen of vragen van ouder(s)/verzorger(s)
- ☐ Anders, namelijk…………

IF beroep = arts, PA

En welke rol(len) zouden kraamverzorgenden volgens uw mening moeten hebben bij pasgeborenen die (in de toekomst) thuis behandeld worden met fototherapie? *Meerdere antwoorden zijn mogelijk*

- ☐ Geen. Kraamverzorgenden zouden hier geen rol in moeten hebben
- ☐ Bloedafname
- ☐ De uitvoering controleren
- ☐ Eerste aanspreekpunt/contactpersoon bij problemen of vragen van ouder(s)/verzorger(s)
- ☐ Anders, namelijk…………

IF beroep = verloskundige

Welke rol(len) zou u als eerstelijns verloskundige willen hebben bij pasgeborenen die (in de toekomst) thuis behandeld worden met fototherapie? *Meerdere antwoorden zijn mogelijk*

- ☐ Geen. Ik heb hier liever geen rol in
- ☐ Ik zou de bloedafname willen doen
- ☐ Ik zou willen controleren of de uitvoering goed wordt gedaan
- ☐ Ik zou graag het eerste aanspreekpunt/contactpersoon willen zijn bij problemen of vragen van ouder(s)/verzorger(s)
- ☐ Anders, namelijk…………

IF beroep = kraamverzorgende

Welke rol(len) zou u als kraamverzorgende willen hebben bij pasgeborenen die (in de toekomst) thuis behandeld worden met fototherapie? *Meerdere antwoorden zijn mogelijk*

☐ Geen. Ik heb hier liever geen rol in
☐ Ik zou willen controleren of de uitvoering goed wordt gedaan
☐ Ik zou graag het eerste aanspreekpunt/contactpersoon willen zijn bij problemen of vragen van ouder(s)/verzorger(s)
☐ Anders, namelijk…………

Deel 6: Kennis en informatievoorziening

Heeft u in de afgelopen 12 maanden (bij)scholing gehad over geelzucht/hyperbilirubinemie bij pasgeborenen?

- o Ja
- o Nee

IF ervaring met fototherapie = ja

U heeft al ervaring met fototherapie thuis. Wij zijn echter benieuwd of u behoefte heeft aan extra informatie over dit onderwerp. Zou u willen aangeven aan welke informatie u behoefte zou hebben? *Meerdere antwoorden zijn mogelijk*

- ☐ Informatie over het herkennen van geelzucht/hyperbilirubinemie
- ☐ Informatie over de gevaren van geelzucht/hyperbilirubinemie
- ☐ Informatie over de voordelen van fototherapie thuis
- ☐ Informatie over de werking van het fototherapie apparaat
- ☐ Informatie over de controles die moeten worden uitgevoerd
- ☐ Informatie over de logistiek (hoe wordt het georganiseerd)
- ☐ Informatie over de financiële vergoeding
- ☐ Geen behoefte aan informatie
- ☐ Anders, namelijk……………………………………

IF ervaring met fototherapie = nee

Het zou kunnen dat fototherapie thuis in de toekomst in uw regio wordt aangeboden aan pasgeborenen met geelzucht/hyperbilirubinemie. We zijn benieuwd waar u in dat geval behoefte aan zou hebben. Zou u willen aangeven aan welke informatie u behoefte zou hebben?

Meerdere antwoorden zijn mogelijk

- ☐ Informatie over het herkennen van geelzucht/hyperbilirubinemie
- ☐ Informatie over de gevaren van geelzucht/hyperbilirubinemie
- ☐ Informatie over de voordelen van fototherapie thuis
- ☐ Informatie over de werking van het fototherapie apparaat
- ☐ Informatie over de controles die moeten worden uitgevoerd
- ☐ Informatie over de logistiek (hoe wordt het georganiseerd)
- ☐ Informatie over de financiële vergoeding
- ☐ Geen behoefte aan informatie
- ☐ Anders, namelijk……………………………………

IF behoefte aan informatie = ja (optie 1 t/m 7 + 9 op vorige 2 vragen)

Hoe zou u deze informatie bij voorkeur aangeboden krijgen?

Meerdere antwoorden zijn mogelijk

- ☐ Voorlichtingsbijeenkomst op locatie (offline)
- ☐ Voorlichtingsbijeenkomst online (bijvoorbeeld via Microsoft Teams of Zoom)
- ☐ E-learning (opgenomen filmpjes en/of opdrachten)
- ☐ Een informatiebrochure
- ☐ Een app
- ☐ Anders, namelijk…..

IF ervaring met fototherapie = nee & behoefte aan informatie = ja

Indien er voorlichtingsbijeenkomsten (offline en/of online) worden georganiseerd, wanneer zou u hier behoefte aan hebben? *Meerdere antwoorden zijn mogelijk*

- ☐ Ruim voor de start van de invoering van fototherapie thuis

☐ Rond de start van de invoering van fototherapie thuis
☐ Ongeveer een maand na de invoering van fototherapie thuis
☐ Ongeveer zes maanden na de invoering van fototherapie thuis
☐ Ongeveer twaalf maanden na de invoering van fototherapie thuis
☐ Ook langer dan een jaar na invoering van fototherapie thuis zou ik behoefte hebben aan regelmatige voorlichtingsbijeenkomsten over het onderwerp

Deel 7: Indicaties en contra-indicaties voor fototherapie thuis

IF beroep = arts, PA

Vanaf welke zwangerschapsduur zouden pasgeborenen die verder gezond zijn volgens uw mening in aanmerking moeten komen voor fototherapie thuis?

- o Vanaf 35 weken
- o Vanaf 36 weken
- o Vanaf 37 weken
- o Vanaf 38 weken
- o Zwangerschapsduur is niet relevant in de beslissing of pasgeborenen in aanmerking komen voor fototherapie thuis

IF beroep = arts, PA

Vanaf hoeveel uur na de geboorte zouden pasgeborenen die verder gezond zijn volgens uw mening in aanmerking moeten komen voor fototherapie thuis?

- o Vanaf 24 uur na de geboorte
- o Vanaf 48 uur na de geboorte
- o Leeftijd is niet relevant in de beslissing of pasgeborenen in aanmerking komen voor fototherapie thuis

IF beroep = arts, PA

Voor welke pasgeborenen is fototherapie thuis volgens u geschikt?

- o Alleen voor pasgeborenen die al thuis zijn en vervolgens hyperbilirubinemie ontwikkelen
- o Ook voor pasgeborenen die op het moment van de vaststelling van hyperbilirubinemie nog in het ziekenhuis verblijven maar waarbij er naast hyperbilirubinemie geen andere indicatie is om nog langer te blijven

IF beroep = arts, PA

Wat zou volgens u de maximale waarde van het serumbilirubine gehalte mogen zijn waarboven fototherapie thuis niet meer aangeboden zou mogen worden?

- o Maximaal 25 µmol/l boven de fototherapiegrens
- o Maximaal 50 µmol/l boven de fototherapiegrens
- o Elke serum bilirubine waarde die onder de wisseltransfusiegrens is
- o Ik denk dat het serumbilirubine gehalte niet relevant is bij de beslissing of fototherapie thuis kan worden aangeboden

IF beroep = arts, PA

Wat zouden volgens u contra-indicaties moeten zijn voor fototherapie thuis?

Meerdere antwoorden zijn mogelijk

- ☐ Laag geboortegewicht en/of dysmatuur
- ☐ Pasgeborenen met een pathologische oorzaak van geelzucht/hyperbilirubinemie, zoals bloedgroepantagonisme
- ☐ Pasgeborenen die volledig borstvoeding krijgen
- ☐ Pasgeborenen waarbij de borstvoeding nog niet volledig op gang gekomen is
- ☐ Pasgeborenen die al fototherapie in het ziekenhuis hebben gehad
- ☐ Een taalbarrière bij de ouder(s)/verzorger(s)
- ☐ Ouder(s)/verzorger(s) die moeite hebben om te begrijpen wat ze moeten doen (door bijvoorbeeld lage gezondheidsvaardigheden)
- ☐ Ouder(s)/verzorger(s) met een lichamelijke beperking of chronische ziekte
- ☐ Ouder(s)/verzorger(s) met een verstandelijke beperking

☐ Ouder(s)/verzorger(s) die al andere kinderen thuis hebben
☐ Alleenstaande ouder(s)/verzorger(s)
☐ Ouder(s)/verzorger(s) die de voorkeur geven voor fototherapie in het ziekenhuis
☐ Twijfel bij de verloskundige of arts of ouder(s)/verzorger(s) in staat zijn om het protocol voor fototherapie thuis te volgen
☐ Anders, namelijk………………

Deel 8: Financiële aspecten en invoering bereidheid

IF ervaring met fototherapie = ja & beroep = arts | verloskundige | PA

Krijgt uw ziekenhuis / uw praktijk op dit moment een financiële vergoeding voor pasgeborenen die thuis worden behandeld met fototherapie?

- o Ja
- o Nee
- o Weet ik niet

IF ervaring met fototherapie = ja & beroep = arts | PA | verloskundige & financiële vergoeding = ja

Is de financiële vergoeding die u op dit moment ontvangt dekkend voor de kosten die gemaakt worden (denk aan personeelskosten, materiaalkosten, kosten bloedonderzoek etc)?

- o Financiële vergoeding dekt de gemaakte kosten volledig
- o Financiële vergoeding dekt de gemaakte kosten grotendeels
- o Financiële vergoeding dekt de gemaakte kosten onvoldoende

IF ervaring met fototherapie = ja

De thuisbehandeling van pasgeborenen met geelzucht/hyperbilirubinemie kost mij extra tijd in vergelijking met de behandeling in het ziekenhuis

Sterk mee oneens	Mee oneens	Neutraal	Mee eens	Sterk mee eens
☐	☐	☐	☐	☐

IF ervaring met fototherapie = ja & beroep = arts | PA, & financiële vergoeding = nee/grotendeels/onvoldoende

Is het gebrek aan financiële vergoeding voor u en/of voor uw ziekenhuis de afgelopen 12 maanden een reden geweest om een pasgeborene die in aanmerking kwam voor fototherapie thuis, toch in het ziekenhuis op te nemen en te behandelen?

- o Nooit
- o Zelden
- o Soms
- o Vaak
- o Altijd

IF ervaring met fototherapie = ja & IF beroep = verloskundige & financiële vergoeding = nee/grotendeels/onvoldoende

Is het gebrek aan financiële vergoeding voor u de afgelopen 12 maanden een reden geweest om niet mee te werken in de zorg voor een pasgeborene die thuis werd behandeld met fototherapie?

- o Nooit
- o Zelden
- o Soms
- o Vaak
- o Altijd

IF ervaring met fototherapie = nee

De volgende twee stellingen hebben betrekking op financiële aspecten van de thuisbehandeling met fototherapie voor geelzucht/hyperbilirubinemie. Kunt u aangeven in welke mate u het eens bent met de stellingen?

IF ervaring met fototherapie = nee

Ik verwacht dat het thuis behandelen van pasgeborenen met geelzucht/hyperbilirubinemie mij extra tijd gaat kosten

Sterk mee oneens	Mee oneens	Neutraal	Mee eens	Sterk mee eens
☐	☐	☐	☐	☐

IF ervaring met fototherapie = nee

Als thuisbehandeling van pasgeborenen met geelzucht/hyperbilirubinemie wordt geïmplementeerd dan vereist dat een financiële vergoeding voor mijn organisatie, praktijk of ziekenhuis

Sterk mee oneens	Mee oneens	Neutraal	Mee eens	Sterk mee eens
☐	☐	☐	☐	☐

IF ervaring met fototherapie = nee & beroep = arts | PA

Indien fototherapie thuis ingevoerd wordt maar er geen of een beperkte financiële vergoeding is, hoe vaak verwacht u pasgeborenen die in aanmerking komen voor fototherapie thuis alsnog op te nemen in het ziekenhuis?

- Nooit
- Zelden
- Soms
- Vaak
- Altijd

IF ervaring met fototherapie = nee & IF beroep = verloskundige

Indien fototherapie thuis ingevoerd wordt maar er geen of een beperkte financiële vergoeding is voor diegene die dan medeverantwoordelijk is voor het leveren van de zorg, hoe vaak zou dat voor u een reden zijn om niet mee te werken in de zorg voor een pasgeborene die thuis wordt behandeld met fototherapie?

- Nooit
- Zelden
- Soms
- Vaak
- Altijd

IF ervaring met fototherapie = nee

Wat zijn voor u en/of uw organisatie (rand)voorwaarden voor het invoeren van fototherapie thuis?

………

………

IF ervaring met fototherapie = nee

Wat hebben u en/of uw organisatie nodig om fototherapie thuis in te voeren?

……

……

IF ervaring met fototherapie = nee

Het laatste deel van de vragenlijst gaat er over in welke mate uw organisatie (uw kraamzorgorganisatie, verloskundigenpraktijk of ziekenhuis) klaar is voor de eventuele invoering van fototherapie thuis. Dit onderdeel bestaat uit 12 stellingen. Kunt u aangeven in hoeverre u het eens bent met deze stellingen?

Collega's van mijn organisatie (kraamzorgorganisatie/verloskundigenpraktijk/ziekenhuis) hebben het vertrouwen dat onze organisatie mensen kan aanzetten om fototherapie thuis in te voeren

Mee oneens	Enigszins mee oneens	Niet mee eens noch oneens	Enigszins mee eens	Mee eens
☐	☐	☐	☐	☐

Collega’s van mijn organisatie (kraamzorgorganisatie/verloskundigenpraktijk/ziekenhuis) zijn bereid zich in te zetten voor de invoering van fototherapie thuis

Mee oneens	Enigszins mee oneens	Niet mee eens noch oneens	Enigszins mee eens	Mee eens
☐	☐	☐	☐	☐

Collega’s van mijn organisatie (kraamzorgorganisatie/verloskundigenpraktijk/ziekenhuis) hebben het vertrouwen dat zij de voortgang van de invoering van fototherapie thuis kunnen volgen

Mee oneens	Enigszins mee oneens	Niet mee eens noch oneens	Enigszins mee eens	Mee eens
☐	☐	☐	☐	☐

Collega’s van mijn organisatie (kraamzorgorganisatie/verloskundigenpraktijk/ziekenhuis) zullen al het mogelijke doen om fototherapie thuis in de praktijk toe te passen

Mee oneens	Enigszins mee oneens	Niet mee eens noch oneens	Enigszins mee eens	Mee eens
☐	☐	☐	☐	☐

Collega’s van mijn organisatie (kraamzorgorganisatie/verloskundigenpraktijk/ziekenhuis) kunnen ondersteunen tijdens de overgang naar fototherapie thuis

Mee oneens	Enigszins mee oneens	Niet mee eens noch oneens	Enigszins mee eens	Mee eens
☐	☐	☐	☐	☐

Collega’s van mijn organisatie (kraamzorgorganisatie/verloskundigenpraktijk/ziekenhuis) willen fototherapie thuis invoeren

Mee oneens	Enigszins mee oneens	Niet mee eens noch oneens	Enigszins mee eens	Mee eens
☐	☐	☐	☐	☐

Collega’s van mijn organisatie (kraamzorgorganisatie/verloskundigenpraktijk/ziekenhuis) hebben het vertrouwen dat zij de urgentie kunnen vasthouden om fototherapie thuis in te voeren

Mee oneens	Enigszins mee oneens	Niet mee eens noch oneens	Enigszins mee eens	Mee eens
☐	☐	☐	☐	☐

Collega’s van mijn organisatie (kraamzorgorganisatie/verloskundigenpraktijk/ziekenhuis) hebben het vertrouwen dat zij om kunnen gaan met uitdagingen die kunnen optreden bij de invoering van fototherapie thuis

Mee oneens	Enigszins mee oneens	Niet mee eens noch oneens	Enigszins mee eens	Mee eens
☐	☐	☐	☐	☐

Collega’s van mijn organisatie (kraamzorgorganisatie/verloskundigenpraktijk/ziekenhuis) zijn vastbesloten fototherapie thuis te in te voeren

Mee oneens	Enigszins mee oneens	Niet mee eens noch oneens	Enigszins mee eens	Mee eens
☐	☐	☐	☐	☐

Collega's van mijn organisatie (kraamzorgorganisatie/verloskundigenpraktijk/ziekenhuis) hebben het vertrouwen dat zij de taken zo kunnen coördineren dat de invoering van fototherapie thuis soepel verloopt

Mee oneens	Enigszins mee oneens	Niet mee eens noch oneens	Enigszins mee eens	Mee eens
☐	☐	☐	☐	☐

Collega's van mijn organisatie (kraamzorgorganisatie/verloskundigenpraktijk/ziekenhuis) zijn gemotiveerd om fototherapie thuis in te voeren

Mee oneens	Enigszins mee oneens	Niet mee eens noch oneens	Enigszins mee eens	Mee eens
☐	☐	☐	☐	☐

Collega's van mijn organisatie (kraamzorgorganisatie/verloskundigenpraktijk/ziekenhuis) hebben het vertrouwen dat ze om kunnen gaan met de beleidsmatige aspecten van invoering van fototherapie thuis

Mee oneens	Enigszins mee oneens	Niet mee eens noch oneens	Enigszins mee eens	Mee eens
☐	☐	☐	☐	☐

Dit is het einde van de vragenlijst.

Hartelijk dank voor het invullen!

We koppelen de uitkomsten van dit onderzoek aan u terug via onze website, onze nieuwsflitsen en via onze webinars.

Mocht u nog vragen hebben, neem dan gerust contact met ons op via regionaalconsortium@hr.nl of m.c.cnossen@hr.nl

S2. **Perception of phototherapy at home among pediatricians, community midwives, and maternity care assistants with and without experience**

Item	Group	Strongly disagree	Disagree	Neutral	Agree	Strongly agree
**I agree that newborns with hyperbilirubinemia should have the possibility to be treated with PT at home**	*Pediatricians and community midwives with experience*	0 (0%)	1 (6%)	0 (0%)	8 (47%)	8 (47%)
	Pediatricians and community midwives without experience	3 (4%)	5 (6%)	10 (11%)	44 (49%)	27 (30%)
	MCAs with experience	1 (2%)	3 (4%)	6 (9%)	27 (40%)	30 (45%)
	MCAs without experience	17 (4%)	41 (9%)	38 (9%)	192 (43%)	158 (35%)
**I think that my colleagues agree that newborns with hyperbilirubinemia should have the possibility to be treated with PT at home**	*Pediatricians and community midwives with experience*	0 (0%)	1 (6%)	2 (12%)	7 (41%)	7 (41%)
	Pediatricians and community midwives without experience	2 (2%)	5 (5%)	13 (15%)	46 (52%)	23 (26%)
	MCAs with experience	0 (0%)	4 (6%)	9 (13%)	40 (60%)	14 (21%)
	MCAs without experience	12 (3%)	27 (6%)	109 (24%)	209 (47%)	89 (20%)
**I think that it is feasible to treat newborns with hyperbilirubinemia with PT at home**	*Pediatricians and community midwives with experience*	0 (0%)	0 (0%)	0 (0%)	9 (53%)	8 (47%)
	Pediatricians and community midwives without experience	3 (3%)	4 (4%)	5 (6%)	54 (61%)	23 (26%)
	MCAs with experience	0 (0%)	4 (6%)	3 (4%)	38 (57%)	22 (33%)
	MCAs without experience	12 (3%)	34 (7%)	32 (7%)	236 (53%)	132 (30%)
**I think that it is worthwhile for my organization to treat newborns with hyperbilirubinemia with PT at home**	*Pediatricians and community midwives with experience*	0 (0%)	1 (6%)	1 (6%)	8 (47%)	7 (41%)
	Pediatricians and community midwives without experience	0 (0%)	7 (8%)	10 (11%)	46 (52%)	26 (29%)
	MCAs with experience	2 (3%)	3 (4%)	8 (12%)	36 (54%)	19 (27%)
	MCAs without experience	15 (3%)	37 (8%)	78 (18%)	199 (45%)	117 (26%)

Legend = items are based on the Team Climate Inventory; questions are completed by 17 pediatricians and community midwives and 67 maternity care assistants (MCAs) with prior experience with phototherapy (PT) at home and 78 pediatricians and community midwives and 446 MCAs without prior experience with PT at home

S3. information needs and information provision

Information needs	*Pediatricians and community midwives with prior experience (n = 15)*	*Pediatricians and community midwives without prior experience (N = 87)*	*MCA with prior experience (n = 57)*	*MCA without prior experience (n = 407)*
Information about recognizing hyperbilirubinemia	*3 (20%)*	*28 (32%)*	*11 (19%)*	*111 (27%)*
Information about the risk of hyperbilirubinemia	*2 (13%)*	*21 (24%)*	*10 (18%)*	*114 (28%)*
Information about the benefits of PT at home	*4 (27%)*	*59 (68%)*	*31 (54%)*	*317 (78%)*
Information about the PT device	*11 (73%)*	*78 (90%)*	*38 (67%)*	*387 (95%)*
Information about controls	*10 (67%)*	*72 (83%)*	*39 (68%)*	*383 (94%)*
Information about logistics	*6 (40%)*	*78 (90%)*	*19 (33%)*	*300 (74%)*
Information about financial compensation	*12 (80%)*	*73 (84%)*	*17 (30%)*	*173 (43%)*
No need for information	*2 (13%)*	*0 (0%)*	*4 (7%)*	*4 (1%)*

How should information be distributed?	*Pediatricians and community midwives with prior experience (N = 13)**	*Pediatricians and community midwives without prior experience (N = 87)*	*MCA with prior experience (n = 53)**	*MCA without prior experience (n = 403)**
In-person information session	*1 (8%)*	*38 (44%)*	*19 (36%)*	*275 (68%)*
Online information session	*8 (62%)*	*62 (71%)*	*15 (28%)*	*136 (34%)*
E learning module	*11 (85%)*	*67 (77%)*	*30 (57%)*	*259 (64%)*
An information brochure	*3 (23%)*	*25 (29%)*	*22 (42%)*	*125 (31%)*
App	*0 (0%)*	*21 (24%)*	*16 (30%)*	*124 (31%)*

**This question was only shown to respondents who indicated to have one or more information needs on the previous question*

Legend = PT = phototherapy; MCA = maternity care assistant

S4. Contraindications phototherapy at home according to physicians (n = 15)

Contraindication	Number (%)
Low birth weight/dysmature	3 (20%)
Pathological cause of hyperbilirubinemia	13 (87%)
Exclusive breastfeeding	0 (0%)
Breastfeeding not yet established	1 (7%)
Prior hospital-based PT	2 (13%)
Language barrier of parents	11 (73%)
Parents with difficulty in understanding the treatment	15 (100%)
Parents with physical disabilities or chronic illnesses	2 (13%)
Parents with intellectual disability	9 (60%)
Other children already at home	0 (0%)
Single parent	0 (0%)
Parents with a preference for hospital care	10 (67%)
Hesitation expressed by midwife or doctor	13 (87%)

Legend: PT = phototherapy

S5. Responses to the Organizational Readiness for Implementation Change (ORIC) questionnaire

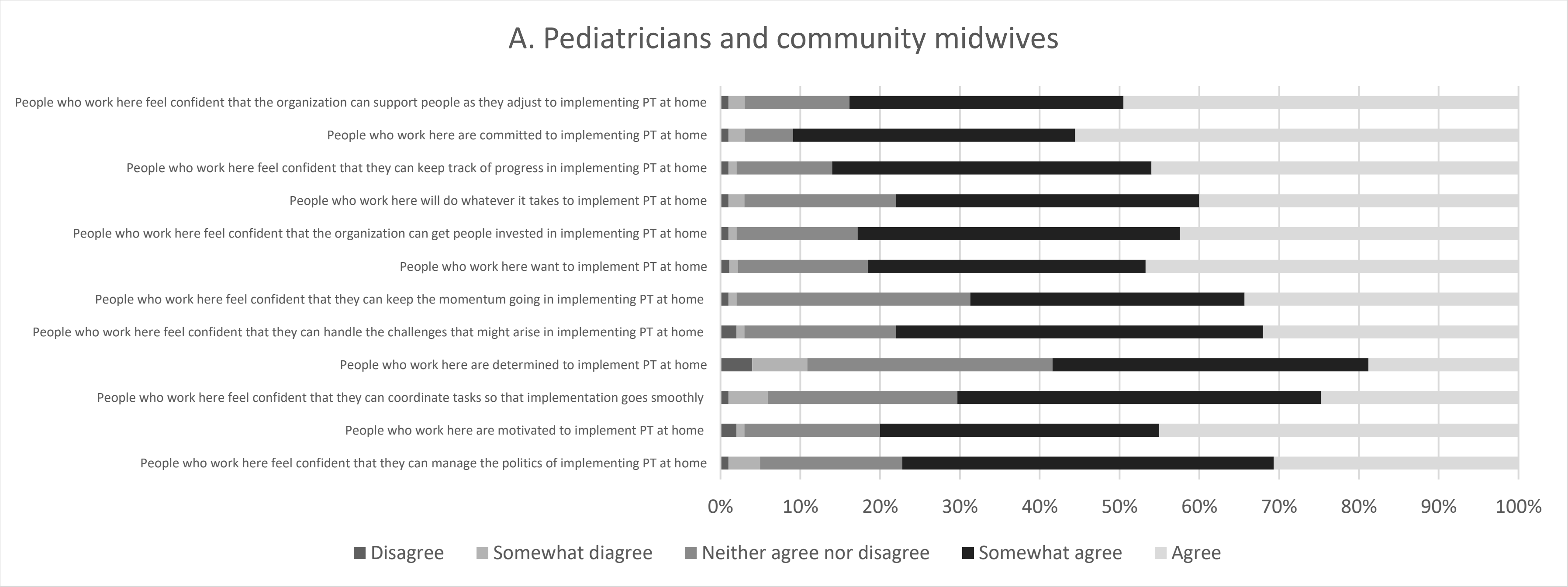

Legend: PT = phototherapy

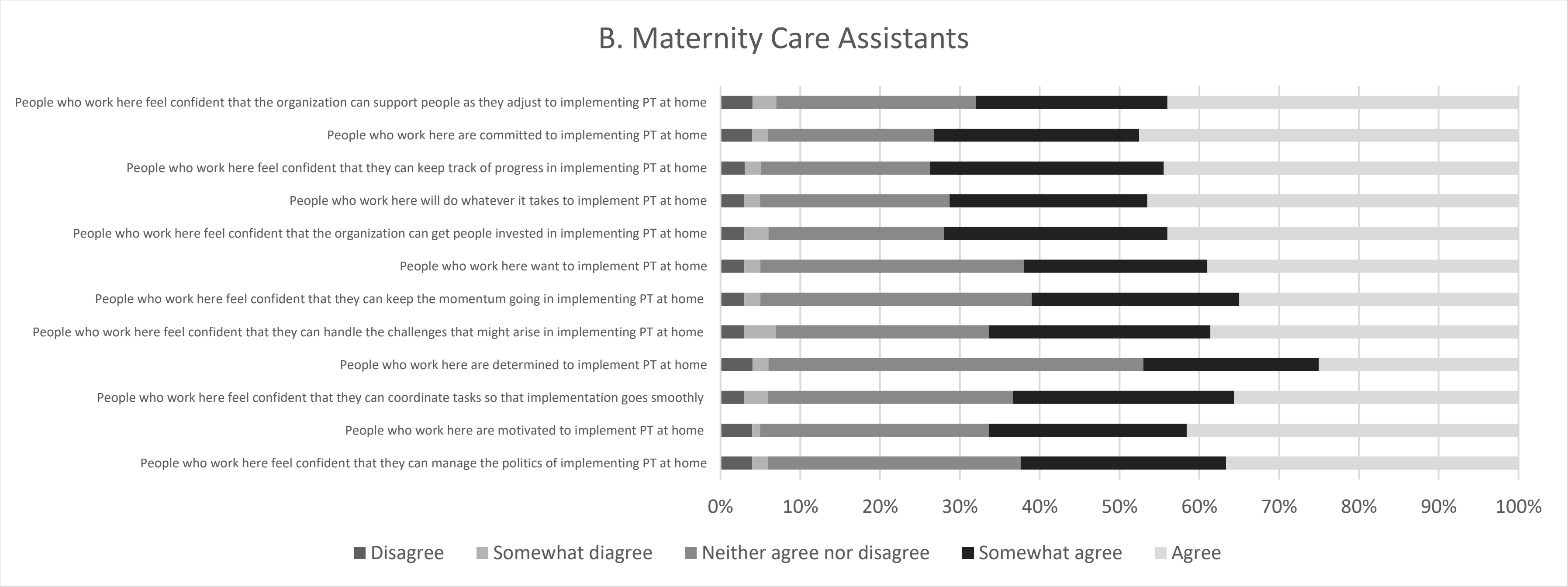

Legend: PT = phototherapy
